# Histopathological findings in colorectal liver metastases after electrochemotherapy

**DOI:** 10.1371/journal.pone.0180709

**Published:** 2017-07-07

**Authors:** Gorana Gasljevic, Ibrahim Edhemovic, Maja Cemazar, Erik Brecelj, Eldar M. Gadzijev, Maja M. Music, Gregor Sersa

**Affiliations:** 1 Department of Pathology, Institute of Oncology Ljubljana, Ljubljana, Slovenia; 2 Department of Surgical Oncology, Institute of Oncology Ljubljana, Ljubljana, Slovenia; 3 Department of Experimental Oncology, Institute of Oncology Ljubljana, Ljubljana, Slovenia; 4 Faculty of Health Sciences, University of Primorska, Izola, Slovenia; 5 Department of Radiology, Institute of Oncology Ljubljana, Ljubljana, Slovenia; 6 Faculty of Health Sciences, University of Ljubljana, Ljubljana, Slovenia; German Cancer Research Center (DKFZ), GERMANY

## Abstract

Electrochemotherapy of colorectal liver metastases has been proven to be feasible, safe and effective in a phase I/II study. In that study, a specific group of patients underwent two-stage operation, and the detailed histopathological evaluation of the resected tumors is presented here. Regressive changes in electrochemotherapy-treated liver metastases were evaluated after the second operation (in 8–10 weeks) in 7 patients and 13 metastases when the treated metastases were resected. Macroscopic and microscopic changes were analyzed. Electrochemotherapy induced coagulation necrosis in the treated area encompassing both tumor and a narrow band of normal tissue. The area became necrotic, encapsulated in a fibrous envelope while preserving the functionality of most of the vessels larger than 5 mm in diameter and a large proportion of biliary structures, but the smaller blood vessels displayed various levels of damage. At the time of observation, 8–10 weeks after electrochemotherapy, regenerative changes were already seen in the peripheral parts of the treated area. This study demonstrates regressive changes in the whole electrochemotherapy-treated area of the liver. Further evidence of disruption of vessels less than 5 mm in diameter and preservation of the larger vessels by electrochemotherapy is provided. These findings are important because electrochemotherapy has been indicated for the therapy of metastases near major blood vessels in the liver to provide a safe approach with good antitumor efficacy.

## Introduction

The most common hepatic neoplasms are metastatic carcinomas predominantly originating from colorectal neoplasms [1]. The majority of patients with colorectal liver metastases have unresectable tumors at the time of diagnosis [2]. Only 10–20% of patients are eligible for surgical resection, which depends on the tumor size as well as the number and position of the metastatic lesions [3]. In patients who are not eligible for major hepatectomy, resection, systemic chemotherapy or a combination of both is performed. The other treatment options for liver metastases are local ablation therapies, such as intratumoral injection of ethanol, radiofrequency ablation or cryotherapy [4].

The new and emerging non-thermal ablative technologies include electrochemotherapy [5] and irreversible electroporation [6,7]. Electrochemotherapy utilizes electroporation for drug delivery, while irreversible electroporation is used in the direct killing of cells. Specifically, electrochemotherapy combines the use of a chemotherapeutic drug, such as bleomycin or cisplatin, with the application of electric pulses as a physical system for facilitated drug delivery to cells [8]. The efficacy on cutaneous metastases is approximately 80% objective response (OR) [9]. However, both electrochemotherapy and irreversible electroporation are also being utilized for the treatment of tumors in internal organs [10–12]. Specifically, our recent clinical pilot study has demonstrated the safety and efficacy of electrochemotherapy in the treatment of colorectal liver metastases with approximately 80% OR [13].

In that first clinical study on electrochemotherapy of colorectal liver metastases, a specific subgroup of patients with good response to treatment were identified with metastases located near or adjacent to major hepatic vessels [13]. In addition, we explored the specific role of electrochemotherapy in the treatment of metastases, which are difficult to treat and are not amenable to surgery or radiofrequency ablation. Furthermore, histological analysis of the treated metastases that were resected afterward demonstrated a good response [13].

The pathological changes in colorectal liver metastases treated using electrochemotherapy were described briefly in that study [13]. So far, the evaluation of responses in most studies have been based on indirect evidence from imaging techniques [11], while the detailed histological evaluation of changes in deep-seated tumors is lacking. Therefore, the aim of this study was to further evaluate the pathological changes in metastases treated using electrochemotherapy by assessing the type of regressive changes, boundary, area of electrode insertion and effect of electrochemotherapy on vascular and biliary structures in the treated areas.

## Materials and methods

### Study design and patients

This study is a prospective, pilot study, conducted at the Institute of Oncology Ljubljana, Ljubljana, Slovenia. Regulatory approvals were obtained from the Institutional Board (Committee for Ethics and Clinical Trials Review of the Institute of Oncology Ljubljana) as well as the National Medical Ethics Committee (#45/09/08). The study is registered at ClinicalTrials.gov: NCT01264952. Written informed consent has been obtained from all patients, which includes the consent to publish the histological images.

A specific subgroup of patients with colorectal liver metastases from our larger, published study on feasibility, efficacy and safety of electrochemotherapy on patients with colorectal liver metastases were included in this study [13]. The baseline demographic characteristics of the study population is summarized in that report [13]. In this histopathological analysis, we included patients from the previous clinical trial, who, due to the extent of their disease, underwent a two‐stage surgery with intent to cure within the standard of care. The second operation was performed 8–10 weeks after the first operation when electrochemotherapy was performed. This two‐stage surgical approach enabled the use of electrochemotherapy during the first operation and tissue collection of metastases for histological analysis during the second operation. The addition of electrochemotherapy did not affect the standard of care given to the patients.

### Electrochemotherapy

Treatment was performed as previously described [10]. Briefly, electrochemotherapy was performed during open surgery by insertion of the electrodes into and around the tumor to cover the whole tumor with a sufficiently intensive electric field with a margin of normal tissue, according to the individualized treatment plan. Electric pulses, generated by Cliniporator (IGEA, Italy) and synchronized with ECG, were delivered after intravenous administration of bleomycin (15,000 IU/m^2^, Heinrich Mack Nachf, GmbH & CO, KG, Illertissen, Germany).

### Tissue processing and evaluation

Tissues for histopathological analysis were available from 7 patients who were operated on twice. Altogether, 13 metastases treated using electrochemotherapy were examined grossly and histologically. The hepatectomy specimens were sectioned into 5-mm slices. The tissues were fixed in 10% buffered formalin for 24 h. Each excised lesion was sampled so that both the central and boundary regions could be examined, including an adjacent rim of non-ablated hepatic parenchyma. The mean number of H&E slides analyzed per tumor was 6 (range 2 to 12 slides). Histological characteristics of the treated areas were analyzed, in particular, regressive changes including necrosis (type and extension), inflammatory infiltrates, changes in blood vessels and biliary ducts in the treated and non-treated areas and eventual residual vital tumor tissue (amount and location). The slides were observed under light microscopy, and representative images from each slide were captured with a DP72 CCD camera (Olympus, Tokyo, Japan) connected to a BX-51 microscope (Olympus) under different magnifications specified in the figure legends (numerical aperture 0.85). Representative images were included in the manuscript.

## Results

### Gross changes

**Fig 1:**
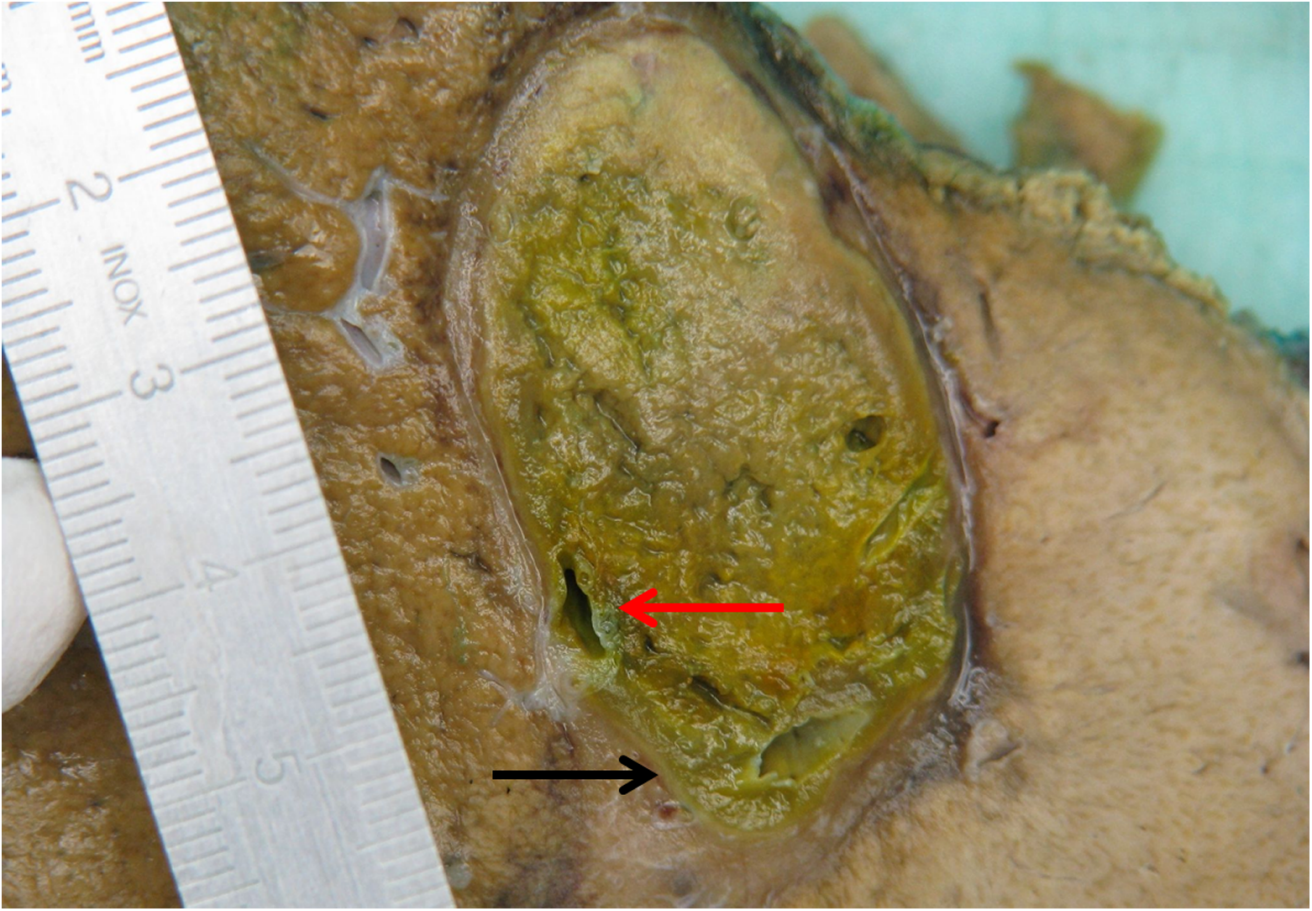
Gross appearance of electrochemotherapy-treated colorectal liver metastasis. The treated area was greenish-yellow. Black arrow indicates the pseudocapsule. Red arrow shows clear lumen of the larger vascular structures.

Macroscopically, the electrochemotherapy-treated areas were quite sharply demarcated from the rest of the liver parenchyma and was primarily oval but focally of somewhat irregular shape. The area was demarcated by a gray pseudocapsule, soft in its consistency and greenish-yellow to brown in color. Some bigger vascular structures measuring >5 mm in diameter with pertinent lumina focally protruded from the ablated area (Fig 1).

### Microscopic changes

#### Boundary of the ablated and normal tissue

**Fig 2:**
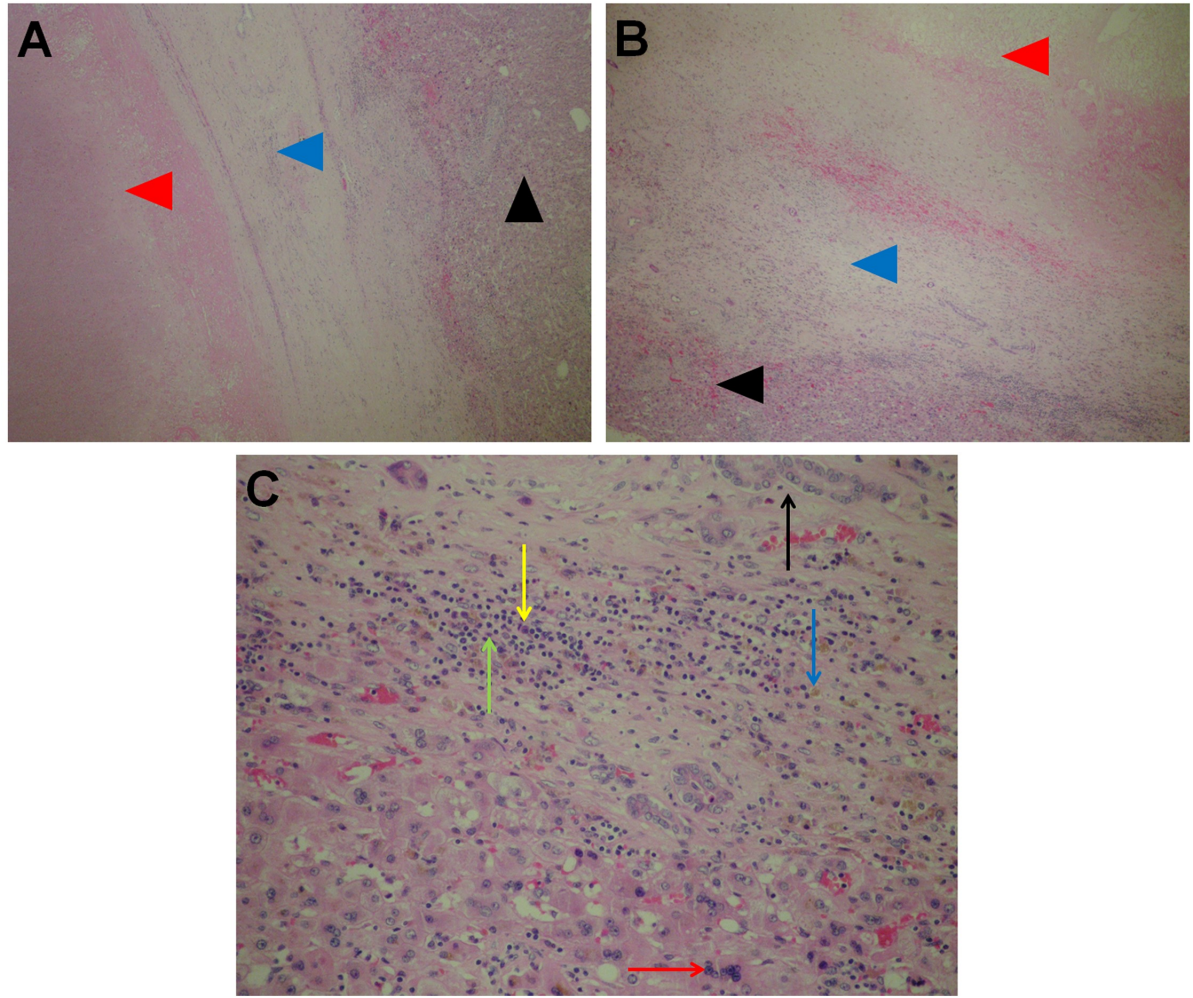
Boundary of the ablated tissue and liver parenchyma. A and B: pseudocapsule (blue arrow heads); necrosis in the ablated area (red arrow heads); viable liver parenchyma outside the treated area (black arrow heads). A: H&E 5x. B: H&E 10x. C: Boundary of the treated hepatic parenchyma and pseudocapsule: some chronic inflammatory infiltrates consisting of lymphocytes (green arrows), plasma cells (yellow arrows), focal siderophages (blue arrows) and proliferating small bile ducts (black arrows) and multinucleated hepatocytes (red arrows). H&E 20x.

Microscopically, there was a band of fibrous tissue at the margin of ablated area and the liver parenchyma; in the fibrous pseudocapsule, focal collections of siderophages were observed along with proliferating small biliary ducts and some smaller blood vessels with diameter up to 0.5 mm (Fig 2A and 2B). On the outer edge of the fibrous tissue, chronic inflammatory infiltrates consisting of lymphocytes and plasma cells were observed in addition to focal multinucleation of hepatocytes (regenerative changes) (Fig 2C). Beyond that region, the liver parenchyma was viable and unremarkable.

#### Ablated area: Area of the electrode insertion

**Fig 3:**
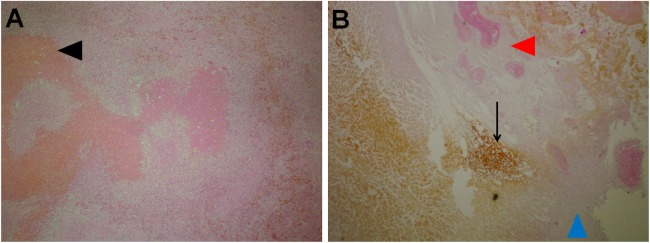
Area of the electrode insertion. A: Tissue areas with necrosis and ingrowth of granulation tissue (black arrow head). H&E 10x. B: Vicinity of the insertional area: complete necrosis of the portal tract (red arrow head) with leakage of bile (black arrow). In the lower right part ingrowth of granulation tissue can be seen (blue arrow head). H&E, 10x.

In the area of ablation, there were traces of electrode insertion (at the periphery as well as in the central parts of the treated area) that were oval, measuring approximately 3–4 mm in the diameter. In these foci, the tissue was completely necrotic, with necrosis of blood vessels and biliary ducts and the presence of biliary lakes in the vicinity of the region of insertion (Fig 3). Some ingrowth of granulation tissue was observed in the described areas.

### Ablated area: Tissue between the electrodes

**Fig 4:**
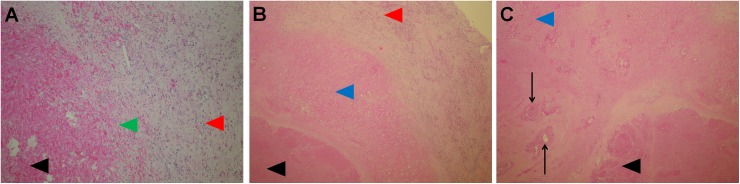
Ablated area. A: Peripheral part of the ablated area with ingrowth of granulation tissue (green arrow head), necrosis (black arrow head), and pseudocapsule (red arrow head). H&E, 10x. B: Coagulation necrosis of the liver parenchyma (blue arrow head), metastatic tumor tissue (black arrow head) and pseudocapsule (red arrow head). H&E, 5x. C: Coagulation necrosis of the liver parenchyma (blue arrow head) with portal tracts (black arrows) and necrotic tumor tissue (black arrow head). H&E, 5x.

The hallmark of the ablated area was coagulation type necrosis (due to obstructed vessels), which was present in the tumor tissue and in the narrow band of liver parenchyma surrounding the metastatic tissue (Fig 4B and 4C). At the peripheral parts of the ablated areas in which coagulation type necrosis was present, proliferation of young fibrous tissue was observed, accompanied with chronic inflammatory infiltrates, foamy macrophages, proliferating capillaries with thin walls and erythrocyte extravasates (Fig 4A).

**Fig 5:**
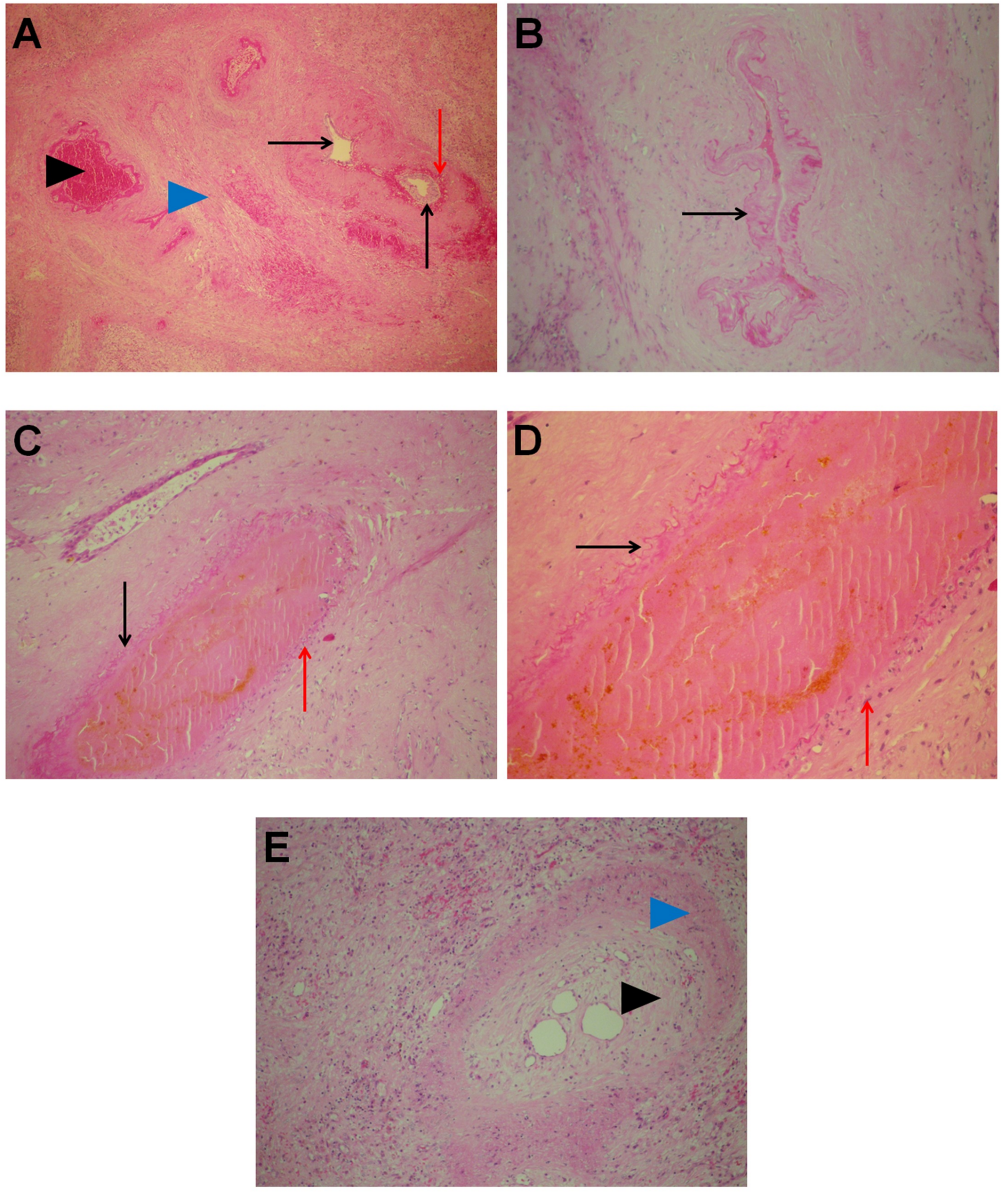
Damage to the liver structures. A: Larger portal tract showing different degrees of damage in the orthotopic structures; biliary duct with intact wall and undamaged epithelium (black arrows). Only some erythrocyte extravasates are present in the walls (red arrow). In the middle and left part of the image, a few blood vessels with various degrees of damage are shown–the branch of the hepatic artery (black arrow head) showing preserved scaffold and the branch of the portal vein showing complete thrombosis (blue arrow head). H&E, 5x. B: Damaged vessel showing preserved structure but without viable cells (preserved scaffold) (black arrow). H&E, 20x. C and D: Different levels of damage in the same blood vessel–on the left portion, there are no viable cells on the vessel wall (black arrows), and only connective tissue structures are present, while the right portion still shows viable cells in the endothelium and tunica media (red arrows). C: H&E 10x; D: H&E, 20x. E: Blood vessel at the periphery of ablated area with recanalized thrombus (black arrow head). Wall of the vessel (blue arrow head). H&E, 20x.

**Fig 6:**
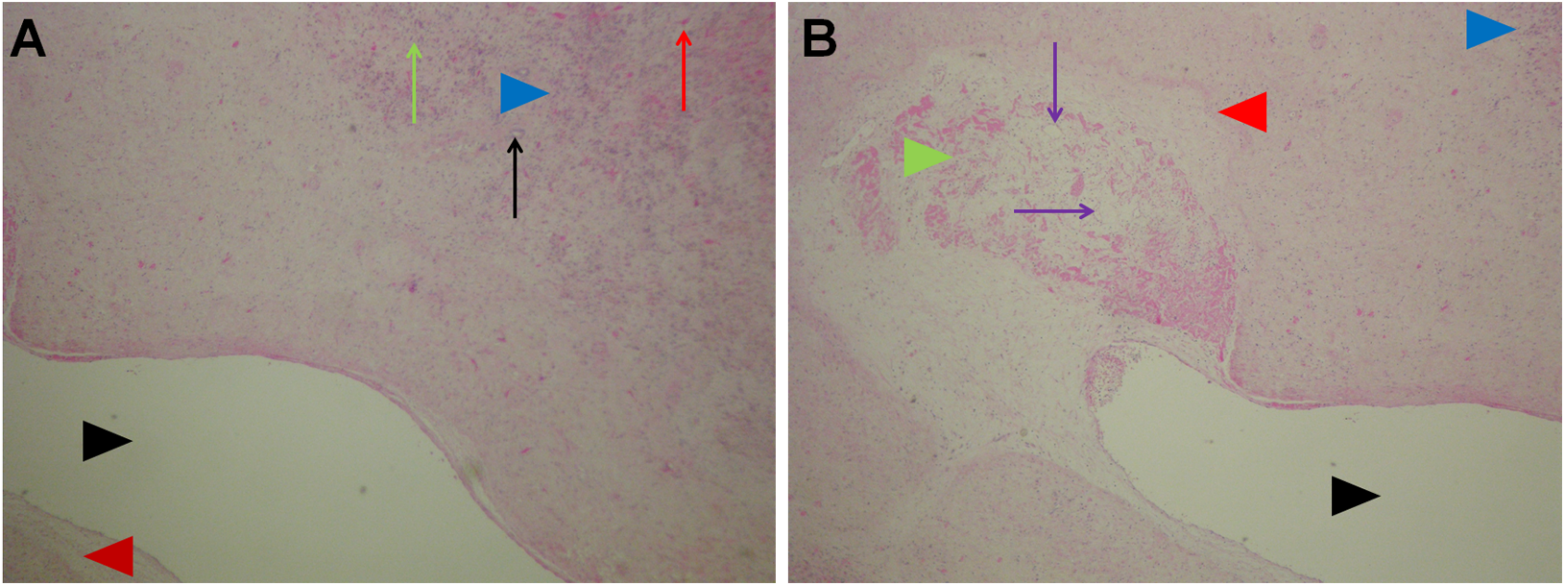
Changes in the vicinity of larger veins. A: Regressive changes extending up to the wall of a large vein without the presence of vital tumor tissue between the vessel wall and area of regressive changes. Lumina of the vein (black arrow head); vessel wall (red arrow head). Area occupied by regressive changes (blue arrow head) with chronic inflammatory cells (green arrow), proliferating small bile ducts (black arrow) and erythrocyte extravasates (red arrow). H&E, 5x. B: Small, peripheral recanalized thrombus (green arrow head) in the same vein located at the very border of the ablated area. Recanalization (purple arrow); venous wall (red arrow head) and lumina (black arrow head). Peripheral portion of the regressive changes is shown in detail in Fig 5A (blue arrow head). H&E, 5x.

**Fig 7:**
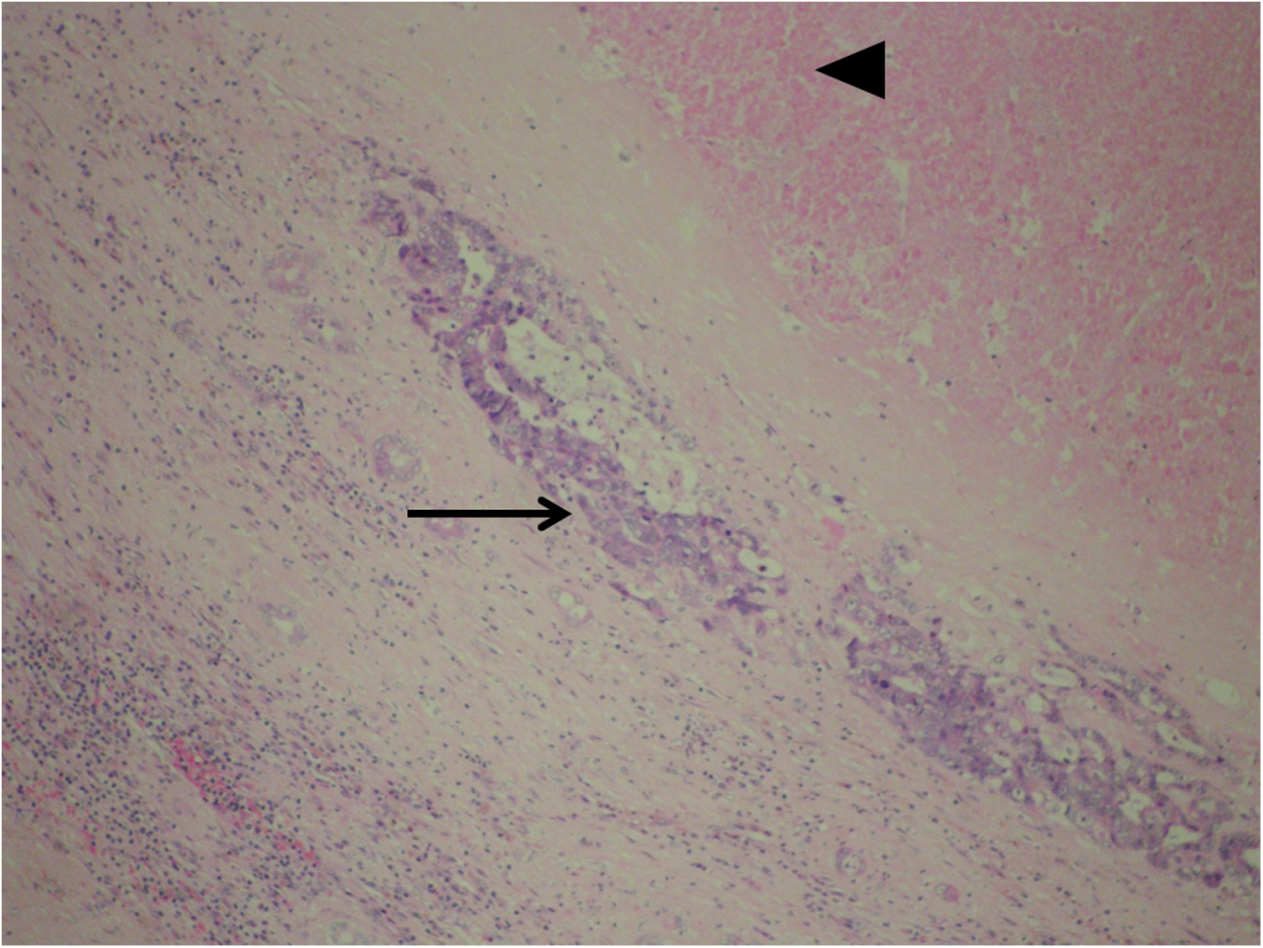
Focus of residual vital carcinoma. Almost completely necrotic tumor tissue (black arrow head). The only focus of the residual vital tumor infiltration is present in the pseudocapsule at the border of ablated area and liver parenchyma (black arrow). H&E, 20x.

The vascular structures of the portal spaces as well as branches of the hepatic veins in the liver parenchyma displayed different changes depending on their size and position in the ablated area: those situated in the central parts of the ablated areas and close to the electrodes showed complete necrosis of all structures (Fig 4C), while those lying farther away from the electrodes displayed different levels of damage. In general, branches of the portal vein showed more pronounced damage than branches of the hepatic artery (Fig 5A). In some of those (mainly the arteries) a pertinent lumina was visible together with the preserved vessel wall, but no viable cells were present in any of the vessel wall layers (preserved scaffold) (Fig 5B). Some other vessels displayed partial damage with part of the vessel wall and endothelial lining being preserved (Fig 5C and 5D), while some exhibited late signs of the endothelial cell damage with thrombosed and recanalized lumina (Fig 5E). Changes of the latter type were seen at the boundary of the treated area in the vicinity of the pseudocapsule. An interesting finding was the presence of regressive changes extending up to the wall of the bigger branches of the hepatic vein without the presence of vital tumor tissue between the vessel wall and area of regressive changes (Fig 6A). In some of those branches, some very small, peripheral recanalized thrombi were present, but those thrombi did not significantly influence the diameter of the blood vessel (Fig 6B). In summary, the functionality of most of the vessels larger than 5 mm in the diameter was preserved, while smaller blood vessels experienced different levels of damage. Analysis of blood vessels in the remaining, non-treated liver parenchyma showed no morphological changes. In the cases without complete pathological response, vital tumor tissue was present mainly at the peripheral parts of ablated areas (Fig 7).

Interestingly, while the vascular structures in the ablated areas showed different levels of damage, the biliary ducts were mainly intact (except in close proximity to the electrodes), with viable cells in the epithelium and pertinent lumina (Fig 5A). In the fibrous tissue of portal spaces with damaged blood vessels, erythrocyte extravasates were present.

## Discussion

This study is the first to demonstrate regressive changes induced in colorectal liver metastases by electrochemotherapy in the liver. Electrochemotherapy induces coagulation necrosis of the treated area containing both tumor and normal tissues. The area became necrotic, encapsulated in a fibrous envelope, and the functionality of most of the vessels larger than 5 mm in diameter and a large proportion of biliary structures was preserved. In contrast, the smaller blood vessels displayed various levels of damage depending on the distance from the electrodes, *i*.*e*., the strength of the electric field. Regenerative changes were already observed in the peripheral parts of the treated area at the time of the observation, *i*.*e*., at 8–10 weeks after the electrochemotherapy.

Electrochemotherapy was initially introduced into clinical practice for the treatment of small cutaneous tumors of different histotypes [14]. Its effectiveness is approximately 80% OR after single treatment. Currently, electrochemotherapy is being introduced for the treatment of tumors larger than 3 cm in diameter and those located in visceral organs [10,11]. In the latter case, the technology is adapted for tumors larger than 3 cm in diameter, with single long needle electrodes inserted centrally and into the normal tissue at the periphery of the tumor. Electric pulses are then delivered between the peripheral and central electrodes to cover the central part of the tumor mass, as well as between the peripheral electrodes in order to ablate the peripheral part of the tumor mass and the safety margin. Due to this complex approach, histological changes need to be explored in detail in order to clarify the mechanisms of action [10].

Electrochemotherapy induces both necrotic and apoptotic cell death, with slow resolution of tumors, based on evidence from preclinical and some clinical histological studies [15]. The histological changes induced in the liver tissue had not been explored in detail until now. In our previous study [13], we obtained indirect evidence of tumor regression, based on radiological evaluation of the tumor response. An 85% complete response rate was observed, including metastases that were located near major blood vessels. Furthermore, histologically, 13 liver metastases that were treated using electrochemotherapy had significantly low amounts of residual vital tumor tissue (9.9 ± 12.2%) compared to the control metastases that were not treated with electrochemotherapy (34.1 ± 22.5%). It is important to note that all these patients were pre-treated with systemic therapy [13].

This study elaborates on the microscopic changes observed after electrochemotherapy in colorectal liver metastases. The typical changes observed were coagulation necrosis with focal hemorrhage. This is expected, since electrochemotherapy has a disruptive effect on vessels, resulting in the destruction of tumor blood vessels [16,17]. This can induce hypoxic conditions and necrotic cell death. Obviously, both the tumor and peripheral liver parenchyma were involved. According to preclinical studies in normal rat liver tissue treated with electrochemotherapy, cell death was also seen on day 14 after the treatment, while on day 56, necrosis was not present any more [18].

The affected area then encapsulates, as seen in the case of radiofrequency ablation [19]. Therefore, the size of the treated lesion cannot be measured as an endpoint to detect response. In such a case, the modified RECIST criteria, which take into account the viability and not the size of the lesion, are more appropriate. This is particularly important for radiological evaluation, at least in the first few months after treatment [20].

Several studies have investigated histological changes in the liver after irreversible electroporation. The studies demonstrated effects of the treatment on small vessels but emphasized that the structure of the bigger vessels remains intact and that after the initial endothelial denudation, its re- epithelization occurs without thrombi formation [21–23]. This is also the case in our study, where we demonstrate that some of the bigger vessels in and around the ablated area remain functional depending on their distance from the electrodes. In general, venules, lacking smooth muscle cells, were more affected than arterioles, indicating that muscle cells can tolerate more damage compared to endothelial cells. These damages to the vessels were especially pronounced in the areas close to the electrodes. With increasing distance from the electrodes, the damage to the vessels decreased. The high sensitivity of endothelial cells to electrochemotherapy was already demonstrated in preclinical studies [24]. Furthermore, certain parts of the vessels affected by electrochemotherapy were without endothelial cell lining, while other parts had such lining. In our previous study, a theoretical model was designed to demonstrate the distribution of the electric field in the vessels according to the position of the vessel in relation to the electric field. The model predicted that vessels perpendicular to the electric field would be exposed to a much greater extent to the field than vessels aligned with it [16]. This could be the reason why some parts of the vessels were found with endothelial cells, while some were not. In addition, re-endothelization could also occur because of preserved blood vessel scaffolds.

In addition, the finite element method for electric field distribution after irreversible electroporation demonstrates that the presence of vascular structures with blood lead to the redistribution of electric fields, leading to areas with more than 60% reduction in the strength of electric field in proximity to large blood vessels and clustered vessel structures. Accordingly, some viable tumor cells were found in the vicinity of large vessels in a rat liver model, while none were seen in the vicinity of small, more isolated vessels [12,25], demonstrating the so called “electric field sinks”. In our patients, such recurrences were not observed, which demonstrated the specific advantage of electrochemotherapy in such clinical cases. The reason for this could be that electrochemotherapy has two components, the electric field and the drug, and much weaker fields are needed for reversible electroporation than for irreversible electroporation. From our histological analysis, it is also evident that no viable tumor cells were present around the larger vessels within the treated area.

An important finding of our study is the observation of preserved biliary structures due to the presence of several layers of fibroblasts and epithelial cells. This finding might have important clinical implications due to the marked decrease in the incidence of biliary complication associated with hepatic tumor ablations, such as bile leakage and biliary fistulas. Other organs could also hypothetically benefit from this property of electrochemotherapy due to the protection conferred to collecting and delivering ducts (pancreas, kidney, prostatic gland, etc.) or the lack of fistula formation.

Furthermore, within the 8–10 weeks of resection of liver lobes and after the electrochemotherapy, already some regenerative changes were observed. The infiltration of the immune cells was present as well as regeneration of the liver parenchyma. We do not have evidence to claim that the liver completely regenerates after electrochemotherapy. However, we are aware of a sporadic case (unpublished data) where hepatocellular carcinoma was treated with electrochemotherapy, and after two years, the treated area completely regressed, so it was not possible to radiologically identify the location of the primary tumor. Such regeneration of the liver with hepatocytes with two nuclei and hepatocytes with large vesicular nuclei was also demonstrated in a preclinical model in rats [26]. However, more studies are needed to confirm these findings.

The histopathological evaluation provided several indications of tumor response to electrochemotherapy. In summary, the findings are as follows:

- In the vicinity of the inserted electrodes, (3–4 mm) tissue damage is observed, which is similar to that seen after irreversible electroporation, with destruction of liver parenchyma, tumor cells, small vessels and bile ducts.
- Due to the vascular disruptive effect of electrochemotherapy and destruction of vascular structures around the electrodes, coagulation necrosis occurs.
- With increasing distance from the electrodes, tumor cell death occurs due to necrosis. In these areas, the vessels larger than 5 mm in diameter are preserved.
- The most sensitive vessels to electrochemotherapy are venules, followed by arterioles, and the most resistant are bile ducts. This is most likely in relation to the thickness of the vessel scaffold and type of cells forming the vessel wall.
- The affected area becomes encapsulated with a fibrotic capsule.
- Around the larger vessels, no viable tumor cells are found.
- Approximately 8–10 weeks after electrochemotherapy, regenerative changes outside the capsule occur with aggregation of immune cells.

In conclusion, electrochemotherapy induces similar changes in the treated area to that seen with irreversible electroporation, *i*.*e*., coagulation necrosis and encapsulation of the treated area. However, some differences may be present concerning the preservation of larger blood vessels and biliary ducts and slow regeneration of the liver parenchyma. These findings may have very important clinical implications regarding complete ablation of inoperable tumors that are in the vicinity of large blood vessels, avoiding complications associated with hepatic tumor ablation as well as with possible complete regeneration of the ablated area. This is important because electrochemotherapy has been indicated for the therapy of tumors in the vicinity of major blood vessels in the liver to provide a safe approach with good antitumor efficacy.
